# Intelligent Algorithm-Assisted Indirect Absorption Spectroscopy for Trace Gas Sensing

**DOI:** 10.3390/s26134054

**Published:** 2026-06-25

**Authors:** Yangkun Huang, Ying He, Shunda Qiao, Haiyue Sun, Yufei Ma

**Affiliations:** 1 National Key Laboratory of Laser Spatial Information, Harbin Institute of Technology, Harbin 150001, China; 17888138289@163.com (Y.H.); shundaqiao@126.com (S.Q.); sunhaiyue282@163.com (H.S.); 2 Zhengzhou Advanced Research Institute, Harbin Institute of Technology, Zhengzhou 450008, China

**Keywords:** photoacoustic spectroscopy (PAS), quartz-enhanced photoacoustic spectroscopy (QEPAS), light-induced thermoelastic spectroscopy (LITES), digital signal processing, machine learning, deep learning, intelligent optimization

## Abstract

Photoacoustic spectroscopy (PAS), quartz-enhanced photoacoustic spectroscopy (QEPAS), and light-induced thermoelastic spectroscopy (LITES) represent indirect absorption spectroscopy techniques for trace gas sensing, whose performance has long been advanced through hardware-oriented enhancement strategies. However, as hardware technologies continue to advance, conventional hardware-based enhancements are increasingly bottlenecked by weak responses, complex cross-interferences, and coupled multiphysics parameters. To transcend these limitations, algorithm-assisted methods, including traditional algorithms, machine learning, deep learning, and intelligent optimization, are being systematically integrated into these spectroscopic systems. This review summarizes recent progress in intelligent indirect absorption spectroscopy from three interconnected dimensions. First, we outline advanced signal processing and spectral reconstruction strategies designed to achieve weak-signal recovery and background noise suppression. Second, the focus shifts to data-driven parameter inversion, showing how multidimensional artificial intelligence models contribute to concentration retrieval, environmental compensation, multicomponent recognition, spectral-overlap decoupling, and front–back-end collaborative waveform coding and demultiplexing. Third, intelligent system optimization is examined, in which surrogate modeling, swarm-intelligence search, physics-guided topology optimization and multi-objective algorithms are employed to improve the design efficiency of the key elements such as photoacoustic resonators and multipass cells (MPCs). Additionally, prospects for future technological developments are also discussed in the concluding section.

## 1. Introduction

Trace gas sensing technologies have been widely applied in environmental monitoring, industrial process control, medical diagnostics, and safety warning [1,2,3,4,5,6,7]. With the increasing demand for high sensitivity, multicomponent selectivity, fast dynamic response, and environmental robustness in practical applications, photonic sensing technologies have been continuously developed [8,9,10,11], among which laser spectroscopic sensing based on molecular “fingerprint” absorption has become an important route for quantitative gas analysis. Compared with conventional electrochemical and semiconductor sensors, which are susceptible to cross-interference, lifetime drift, and environmental perturbations, laser spectroscopic techniques offer intrinsic molecular selectivity, potential for non-contact measurement, and capability for in situ and real-time online monitoring, showing distinct advantages in trace gas detection under complex conditions [12,13,14,15,16,17,18,19,20,21,22].

Among various laser spectroscopic methods, photoacoustic spectroscopy (PAS), quartz-enhanced photoacoustic spectroscopy (QEPAS), and light-induced thermoelastic spectroscopy (LITES) constitute a representative class of indirect absorption-based laser spectroscopic techniques [23,24,25,26,27,28,29,30]. Conventional direct absorption spectroscopy generally relies on photodetectors to measure the attenuation of transmitted optical intensity, and its performance can be limited by detector response range, thermal noise, cooling requirements, and system cost, especially in the mid-infrared, far-infrared, and terahertz regions where strong fundamental molecular absorption bands are located. In contrast, indirect absorption-based spectroscopy does not directly read out transmitted optical intensity. Instead, the absorbed optical energy is converted into acoustic waves, piezoelectric responses, or localized thermoelastic vibrations, which are subsequently detected by microphones or high-quality-factor quartz tuning forks (QTFs). This energy-conversion mechanism can alleviate the wavelength-response and cost limitations of photodetectors while enhancing weak absorption signals through acoustic resonance or high-Q mechanical response. Since Kosterev et al. introduced QEPAS in 2002 and Ma et al. proposed LITES in 2018 [31,32], these techniques have continuously expanded their application boundaries in environmental noise immunity, miniaturization, non-contact detection, and sensing under complex operating conditions.

**Figure 1:**
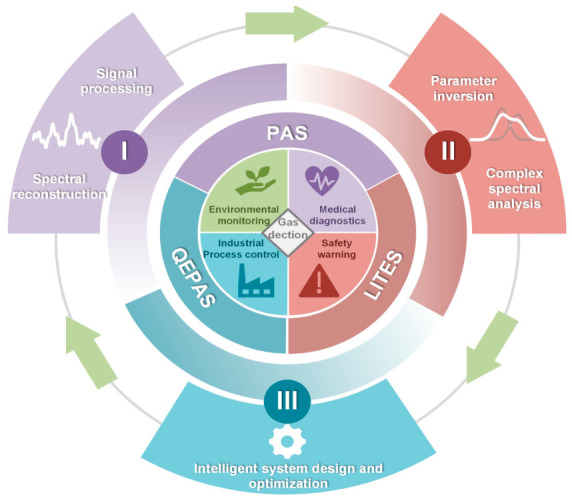
Conceptual framework of intelligent algorithm-assisted PAS-, QEPAS-, and LITES-based trace gas sensing.

For a long time, performance enhancement in PAS, QEPAS and LITES system has mainly relied on hardware-oriented strategies, such as high-power excitation, advanced light sources, customized QTFs, acoustic resonance amplification, multipass cell (MPC) configurations, and fast demodulation [33,34,35,36,37,38,39,40,41]. As these hardware approaches continue to mature, however, further improvement is increasingly limited by factors that cannot be solved by hardware scaling alone. Weak absorption signals are easily buried by random noise, baseline drift, optical fringes, and electronic fluctuations, leading not only to signal-to-noise ratio (SNR) degradation but also to distortion of the absorption-related spectral morphology. Moreover, temperature and pressure variations, nonlinear sensor responses, and multicomponent cross-interference can reduce the accuracy of concentration inversion and make gas recognition and component decoupling more difficult. At the device-design level, the performance of PACs, acoustic resonators, QTFs, and MPCs is governed by strongly coupled structure–response parameters, including cavity geometry, acoustic-mode distribution, QTF coupling position, optical path length, incident angle, mirror spacing, and spot-pattern regularity; therefore, empirical design and one-factor parameter sweeping are often inefficient. These bottlenecks indicate that laser spectroscopic sensing requires algorithm-assisted strategies to move beyond conventional hardware enhancement and empirical calibration. In this context, traditional signal-processing algorithms, machine learning, deep learning, and intelligent optimization methods have been introduced to improve signal reliability, quantitative robustness, interference resistance, and sensor-design efficiency. Accordingly, this review organizes recent progress into three interconnected directions: signal processing and spectral reconstruction, parameter inversion and complex spectral analysis, and intelligent system design and optimization, forming the conceptual framework shown in Figure 1. Specifically, signal processing and spectral reconstruction address the degradation of measured responses and recover more reliable spectral features; parameter inversion and complex spectral analysis convert these features into quantitative concentration, component, and interference-decoupled information; and intelligent system design and optimization improve the front-end structures that determine the original signal strength, stability, and information quality.

## 2. Fundamentals of Photoacoustic and Thermoelastic Laser Spectroscopy

**Figure 2:**
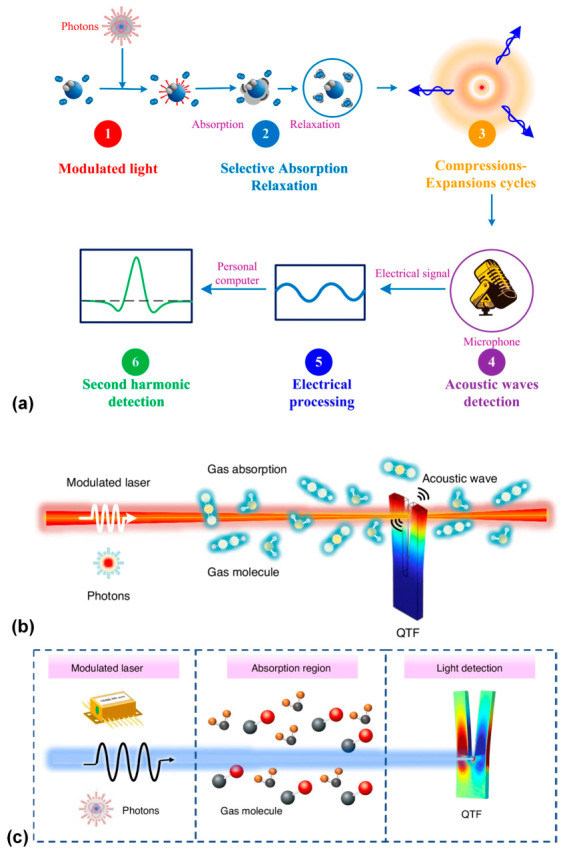
The principle of different laser spectroscopy: (**a**) PAS [12]; (**b**) QEPAS [13]; and (**c**) LITES [13]. (**a**) is reproduced from ref. [12] with permission from MDPI by CC BY 4.0. (**b**,**c**) are reprinted from ref. [13] with permission from Springer Nature by CC BY 4.0.

The principle of different laser spectroscopy is shown as Figure 2. In PAS, modulated laser absorption is converted into periodic heat release through non-radiative relaxation, generating pressure oscillations that are detected by a microphone or acoustic transducer. Under fixed gas and cell conditions, the PAS signal can be qualitatively expressed as:

(1) $$S_{PAS}\propto P_{0}\alpha \left(\nu \right)CQ$$

where *P*_0_ is the optical power, *α*(*ν*) is the absorption coefficient, *C* is the gas concentration, and *Q* denotes the acoustic enhancement factor. This relationship shows that PAS performance is strongly affected by PAC geometry, resonance frequency, and acoustic-mode distribution, making acoustic-cell and resonator design suitable targets for simulation-assisted and algorithm-guided optimization. QEPAS follows the same photoacoustic conversion process, but uses a QTF instead of a microphone to detect acoustic excitation through piezoelectric transduction. When the acoustic excitation matches the QTF resonance, the QEPAS signal can be described as:

(2) $$S_{QEPAS}\propto P_{0}\alpha \left(\nu \right)CQ$$

where *P*_0_, *α*(*ν*), and *C* have the same meanings as in Equation (1), *Q* is the QTF quality factor and *η* represents the acoustic coupling efficiency. Therefore, QEPAS performance depends on molecular absorption, QTF resonance characteristics, acoustic micro-resonator geometry, and acoustic-field–QTF coupling. LITES differs from QEPAS because the QTF is excited by light-induced thermoelastic vibration rather than gas-generated acoustic waves. The absorption-modulated transmitted beam is focused onto the QTF, where periodic optical heating induces thermoelastic expansion and piezoelectric readout. Its signal can be qualitatively written as:

(3) $$S_{LITES}\propto P_{t}Q\eta ,\;P_{t}\propto P_{0}\alpha \left(\nu \right)CL$$

where *P_t_* is the transmitted optical power incident on the QTF, *P*_0_, *α*(*ν*)*,* and *C* have the same meanings as in Equation (1), *Q* is the QTF quality factor, *L* is the effective optical path length and *η* denotes the thermoelastic conversion efficiency. LITES benefits from multipass optical-path enhancement, but dense ray trajectories, spot distributions, and alignment feasibility introduce coupled optical-design parameters, making algorithm-assisted MPC optimization increasingly important. QTF geometry, resonance frequency, prong spacing, and excitation position also strongly affect QEPAS and LITES performance. However, compared with PAC and MPC optimization, algorithm-guided QTF and coupling-structure optimization remains much less explored.

In practical measurements, the recorded harmonic signal is not the ideal absorption response alone, but a composite result of molecular absorption, background interference, and random noise. For wavelength-modulation-based PAS, QEPAS, and LITES, the measured signal can be generally expressed as:

(4) $$S_{measured}\left(x\right)=S_{absorption}\left(x;\theta \right)+B\left(x\right)+n\left(x\right)$$

where x denotes time, frequency, wavelength, or spectral sampling point, and $S_{absorption}\left(x;\theta \right)$ represents the absorption-related sensing response determined by gas concentration, environmental parameters, and system response. $B\left(x\right)$ denotes slowly varying backgrounds, optical fringes, or baseline distortions, while $n\left(x\right)$ represents random noise. Therefore, signal processing and spectral reconstruction aim to recover $S_{absorption}$ from the measured signal, whereas parameter inversion and complex spectral analysis aim to estimate gas concentration or component composition while compensating for environmental interference. Although PAS, QEPAS, and LITES differ in transduction pathways, their intelligent development can therefore be commonly organized into three interconnected branches: signal processing and spectral reconstruction, parameter inversion and complex spectral analysis, and intelligent system design and optimization. Within this common framework, algorithmic adaptation should still reflect the distinct noise and response characteristics of each technique: PAS is mainly affected by acoustic background and microphone/electronic noise, QEPAS is strongly related to QTF resonance and acoustic coupling, whereas LITES is more sensitive to transmitted-power fluctuation, optical fringes, and thermoelastic excitation conditions. Therefore, algorithm selection and model inputs should be adjusted according to the dominant interference source and sensing mechanism of each system.

## 3. Signal Processing and Spectral Reconstruction

In PAS-, QEPAS-, and LITES-based sensing, the measured harmonic signal is usually a degraded response affected by weak absorption intensity, background absorption, random noise, optical baseline fluctuation, electronic interference, acoustic disturbance, and system drift. Therefore, signal processing should not be understood as simple smoothing, but as the recovery of a quantitatively usable spectral response. Early studies mainly relied on traditional signal processing algorithms. In broadband differential infrared PAS, Liu et al. combined differential photoacoustic detection with wavelet-domain denoising: the differential configuration suppressed coherent noise, optical baseline contributions, and overlapped background absorption, while multiscale wavelet shrinkage further reduced residual incoherent interference [42]. For QEPAS signals whose useful response overlaps with noise in the frequency domain, Xie et al. introduced stochastic resonance, using a monostable nonlinear system to convert an appropriate level of noise into weak-signal enhancement [43]. For online QEPAS detection, Zhou et al. used adaptive Kalman filtering to update filtering behavior under changing measurement conditions, reducing the dependence on manually selected noise parameters [44]. In CH_4_-LITES, Liu further introduced an adaptive Savitzky–Golay algorithm with a χ^2^ statistical criterion, so that random fluctuations could be suppressed while the main 2*f* morphology was retained [45]. As another decomposition-based route, T. Zhang et al. proposed an SVMD-PE-SG method for CH_4_-QEPAS 2*f* denoising, where successive variational mode decomposition separates the measured harmonic signal into modal components, permutation entropy is used to select signal-preserving components for reconstruction, and SG filtering further smooths the reconstructed response. In general, wavelet- and decomposition-based methods are suitable for multiscale noise suppression, Kalman filtering is more appropriate for dynamic or time-varying measurements, and SG-type smoothing is useful when the main spectral morphology needs to be preserved with low computational complexity. These traditional and adaptive methods are valuable because they are interpretable and easy to deploy, but their performance still depends on signal assumptions, parameter selection, and noise-state estimation [46].

**Figure 3:**
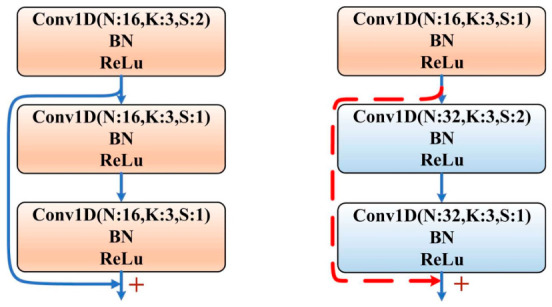
The structure of residual convolutional blocks [47]. It is reproduced from ref. [47] with permission from Elsevier.

Learning-based methods provide a more flexible route when noise and interference become nonlinear, mixed, or difficult to describe by predefined filtering rules. Cao et al. introduced a deep residual network for PAS methane signal filtering and concentration retrieval, which is shown in Figure 3. The key idea is residual learning: under supervised training, the model learns the mapping from noise-contaminated 2*f* signals to reference clean-label 2*f* signals, rather than relying on fixed frequency bands, manually selected thresholds, or local smoothing windows. The absorption-related harmonic profile is already embedded in the measured waveform, while the algorithm mainly needs to remove noise and interference without erasing intrinsic spectral morphology. Residual connections help retain useful low-level spectral information during deep feature mapping, reducing the risk of signal degradation and over-smoothing. The method substantially improved SNR at multiple CH_4_ concentrations and achieved an MDL of 1.47 ppb [47].

**Figure 4:**
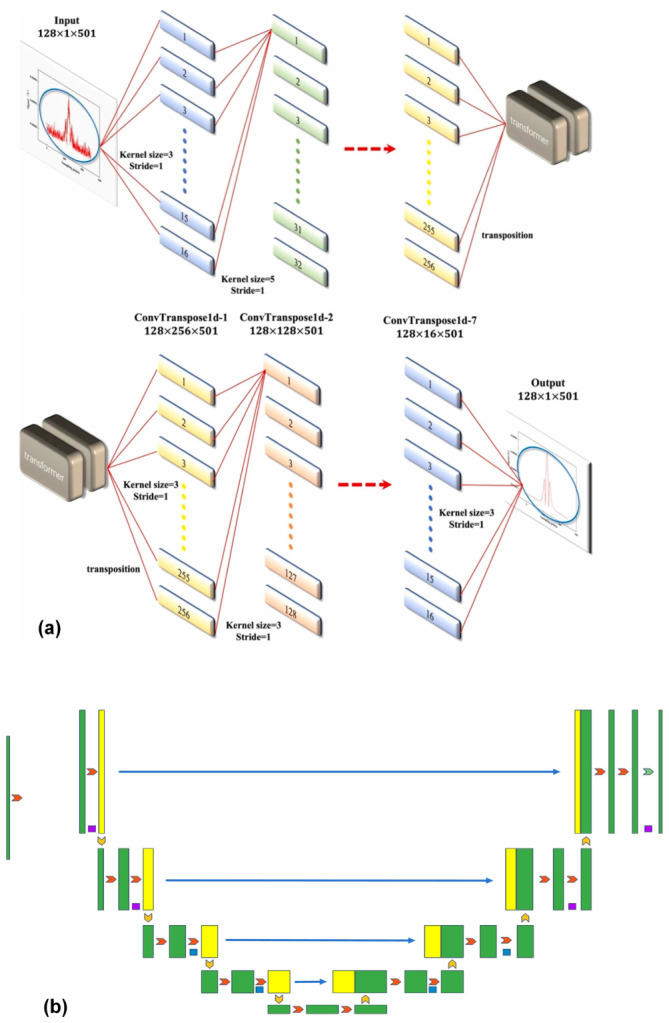
(**a**) CNN–Transformer framework for local–global 2*f* spectral reconstruction [48]. (**b**) Representative 1D U-Net encoder–decoder architecture for 2*f* denoising. (**a**) is reproduced from ref. [48] with permission from Elsevier.

When low-concentration harmonic signals are corrupted by more complex mixed noise, deep reconstruction models provide a more flexible solution. Zhang et al. proposed a CNN–Transformer framework for differential resonant PAS shown as Figure 4a: the CNN component extracts local 2*f* features such as peak edges, valleys, and side-lobe variations, while the Transformer component captures long-range dependencies across the full harmonic profile, so denoising is performed as local–global spectral reconstruction rather than ordinary waveform smoothing. The CNN–Transformer framework improved the SNR of 500 ppb C_2_H_2_ signals by 70 times [48]. As a complementary encoder–decoder route, Xiao et al. applied a 1D U-Net to QEPAS 2*f* traces, where multiscale encoding and progressive decoding reconstruct the denoised spectrum, and skip connections transfer shallow waveform details to reduce peak-shape loss. Figure 4b provides a representative schematic illustration of the U-Net architecture. The 1D U-Net improved the SNR of H_2_O signals by 2.05 times and achieved an MDL of 2.21 ppm at 619 s [49]. Similar U-shaped reconstruction has also been extended to high-sensitivity QEPAS and PAS sensing. Wang et al. integrated a U-Net-based neural noise filter with a clamp-type QTF, dual-tube acoustic micro-resonator, and EDFA-enhanced HCN-QEPAS system, showing that encoder–decoder reconstruction can improve weak 2*f* signal recovery while preserving peak morphology [50]. Cheng et al. further introduced U-Net++ into a rollar-type resonator PAS system for CH_4_ sensing, where nested dense skip pathways and deep supervision were used to reduce the semantic gap between encoder and decoder features and enhance spectral feature reuse during reconstruction [51].

**Figure 5:**
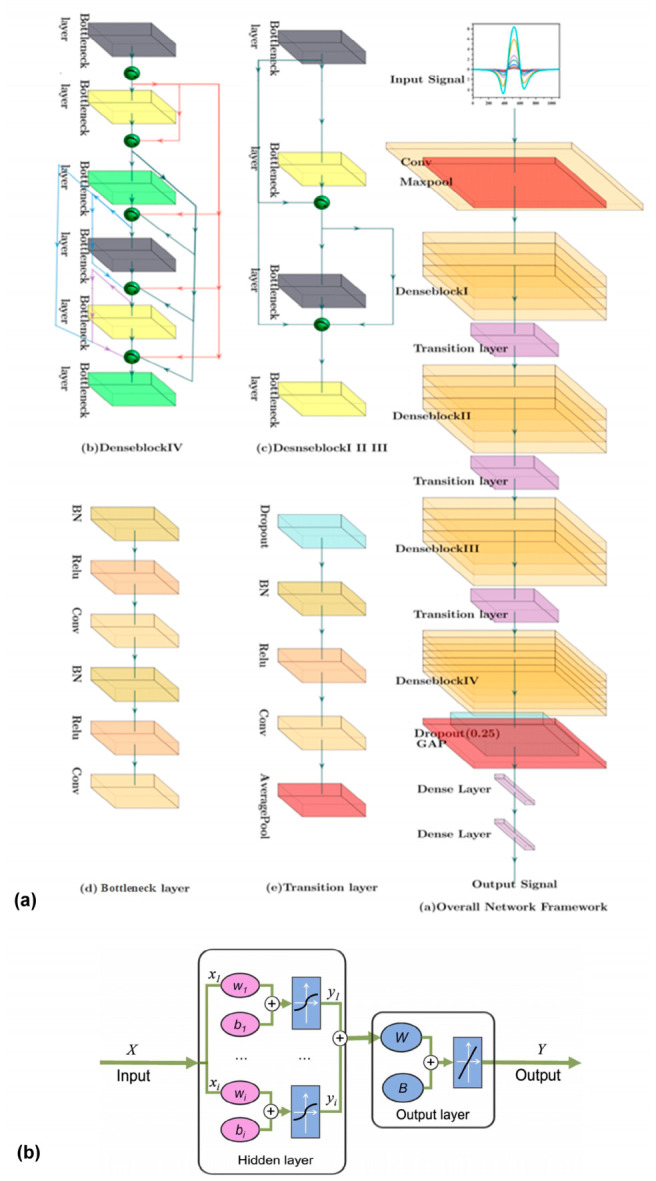
(**a**) DenseNet-based correction model for structured fringe suppression [52]; (**b**) SNN fitting model for lightweight spectral denoising [53]. (**a**) is reproduced from ref. [52] with permission from Elsevier. (**b**) is reproduced from ref. [53] with permission from Elsevier.

Structured interference requires additional correction because it is not random noise and may overlap with absorption-related harmonic features. In multipass-enhanced PAS, inter-component and intra-component etalon effects from lenses, mirrors, windows, and resonators can modulate the transmitted laser intensity and generate fringe-like spectral distortion. Cao et al. addressed this problem with a DenseNet-based correction model. Figure 5a demonstrates each dense block in DenseNet reuses feature maps from all preceding layers, so shallow fringe-related patterns and deeper absorption-related features can be jointly represented; transition layers then compress the feature dimension, and the final layers reconstruct the corrected spectrum [52]. This dense feature reuse improves the separation between structured fringes and useful absorption signals while maintaining spectral fidelity. In another lightweight learning-based route, Liu introduced shallow neural-network fitting into HF-LITES spectral processing, as shown in Figure 5b. The SNN directly takes scanned spectral data as the input and learns a smooth fitted response, providing a compact neural denoising strategy for spectra affected by interference fringes, baseline shifts, and random noise without complex preprocessing. DenseNet increased the SNR from 7 to 266 at 5 ppm CH_4_ and achieved an MDL of 0.226 ppb at 494 s, while SNN fitting improved the SNR by 2.0 times and achieved an MDL of 71 ppb at 110 s [53].

In summary, signal processing and spectral reconstruction have moved from traditional signal-processing algorithms toward more efficient and higher-performance AI-based adaptive signal recovery. Traditional methods remain effective for weak-signal extraction, dynamic stabilization, and morphology-preserving smoothing, but they are often limited by empirical parameter selection and assumptions about noise behavior. Deep-learning methods further improve the flexibility of signal recovery by learning mappings from degraded measurements to reference spectral responses. Compared with traditional filtering methods, residual networks, encoder–decoder models, Transformer-based architectures, dense feature-reuse networks, and lightweight SNN-based fitting models are more suitable for nonlinear, mixed, or structured interference, but they generally require representative training data and, except for shallow models, higher computational resources.

## 4. Parameter Inversion and Complex Spectral Analysis

### 4.1. Concentration Retrieval and Multi-Parameter Decoupling

**Figure 6:**
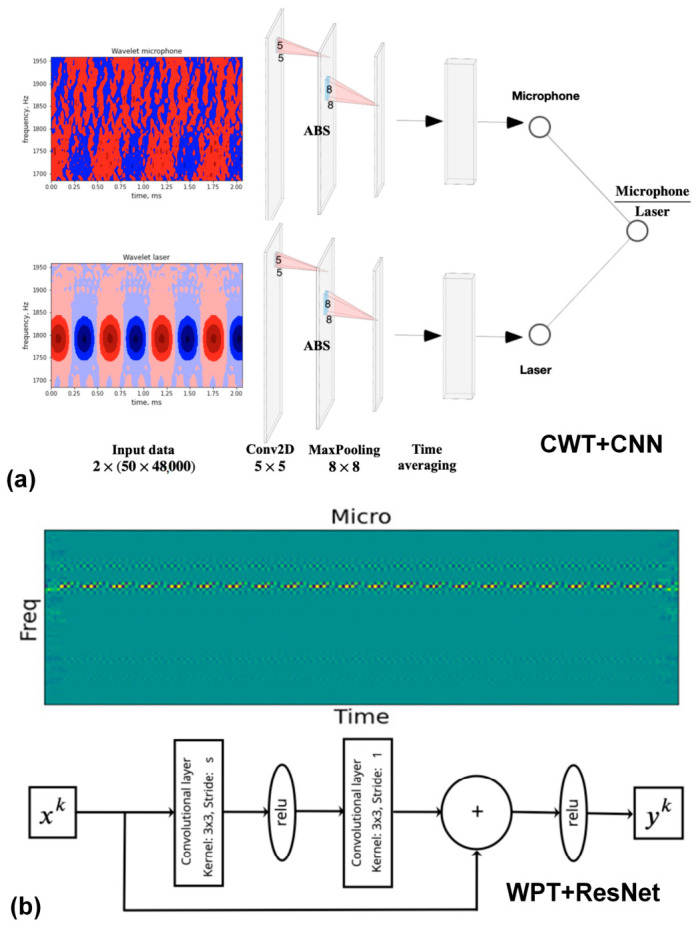
Wavelet-assisted dual-channel learning architectures: (**a**) CWT-CNN [54] and (**b**) WPT-ResNet [54]. (**a**,**b**) are produced from ref. [54] with permission from MDPI by CC BY 4.0.

After signal processing and spectral reconstruction improve the fidelity of measured responses, the next challenge is to transform signals into reliable quantitative sensing information. Concentration retrieval becomes unreliable when weak signals, laser source fluctuations, nonlinear sensor responses, and environmental perturbations distort the relationship between measured spectra and gas concentration. Under low-SNR conditions, wavelet-assisted dual-channel acoustic–optical representation learning provides a useful route by jointly exploiting the microphone signal and the laser reference signal before regression. Kozmin et al. evaluated this idea for photoacoustic methane sensing [54]. The microphone channel contains the absorption-induced acoustic response, while the laser channel records source-intensity variations; therefore, their joint use introduces a multimodal sensing perspective in which the model learns not only the gas-induced response but also the optical-source fluctuation that modulates it. Figure 6 illustrates two routes. In the continuous wavelet transform (CWT)-based route, the two synchronized time-domain signals were transformed into large wavelet time–frequency maps, allowing a CNN to learn joint acoustic–optical features beyond the single resonance-amplitude ratio used in conventional FFT analysis. However, the high dimensionality of the CWT maps increased computational cost and limited model efficiency. Therefore, the authors further introduced wavelet packet transform (WPT)-based representations, where both microphone and laser traces were decomposed into compact multiscale sub-band maps. These WPT maps were then processed by CNN-type regressors, including VGG- and ResNet-based architectures, to learn concentration-related features from the paired acoustic–optical inputs. Compared with FFT-based amplitude extraction and CWT-CNN, the WPT–ResNet route achieved the best low-concentration prediction, reducing the MSE and MAPE to 0.011 ppm^2^ and 5.4% at 1.9 ppm CH_4_ under PSNR = 5.47 dB.

**Figure 7:**
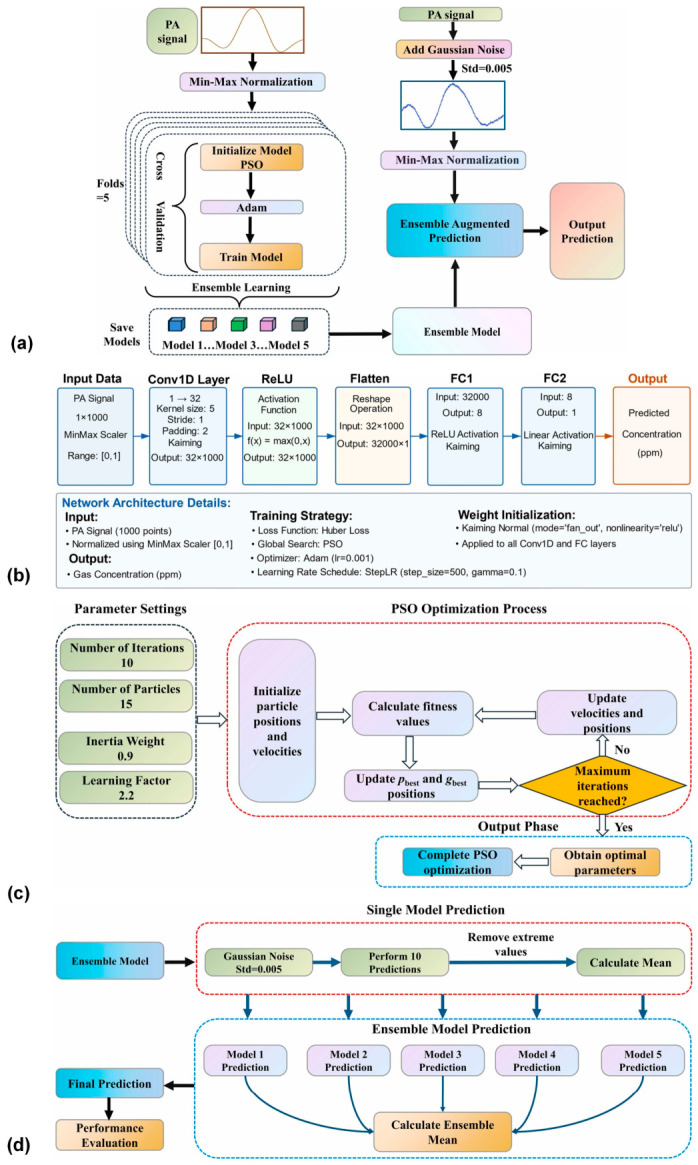
Schematic diagram of PSO-EAP-CNN: (**a**) ensemble-learning and augmented-prediction workflow [55]; (**b**) CNN regression architecture [55]; (**c**) PSO-based parameter optimization process [55]; and (**d**) ensemble augmented prediction strategy [55]. (**a**–**d**) are reproduced from ref. [55] with permission from Elsevier.

Different from feature-representation optimization, Su et al. addressed regression robustness in PAS concentration prediction using a PSO-EAP-CNN framework, as shown in Figure 7 [55]. The CNN first extracts waveform-level features from the normalized PA signal and maps them to concentration values through a regression output. PSO is then used to optimize the CNN weights and biases before gradient-based training, reducing the risk of local optima and improving training stability. In addition, five base CNN models are trained through cross-validation and integrated into an ensemble model; during prediction, the EAP strategy introduces controlled Gaussian perturbations, performs repeated predictions, removes extreme values, and averages the remaining outputs. This design directly targets noise-induced prediction variance and correlation degradation rather than only improving spectral SNR. The final PSO-EAP-CNN reduced the MAE, RMSE, and MAPE by 43.76%, 39.25%, and 51.15% compared with the baseline CNN, and by 68.55%, 67.43%, and 75.21% compared with OLS.

Beyond noise-induced regression errors, practical measurements are also affected by environmental perturbations, especially temperature-induced changes in gas density, sound velocity, and photoacoustic signal propagation. Sun et al. addressed this issue in mixed-gas PAS detection by combining gas classification with temperature compensation [56]. The KNN-SVM module improves qualitative gas identification by using KNN to reclassify ambiguous samples near the SVM hyperplane, thereby improving classification robustness for mixed gases. For quantitative correction, the WOA-BP model uses whale optimization to search for a better BP neural-network structure and parameters, and then learns the nonlinear relation between temperature-dependent photoacoustic responses and corrected gas concentration. The KNN-SVM classifier achieved 99.167% accuracy and 99.375% AUC, while the WOA-BP compensation model achieved R^2^ = 97.89%, MAE = 1.4868, and RMSE = 2.0416.

**Figure 8:**
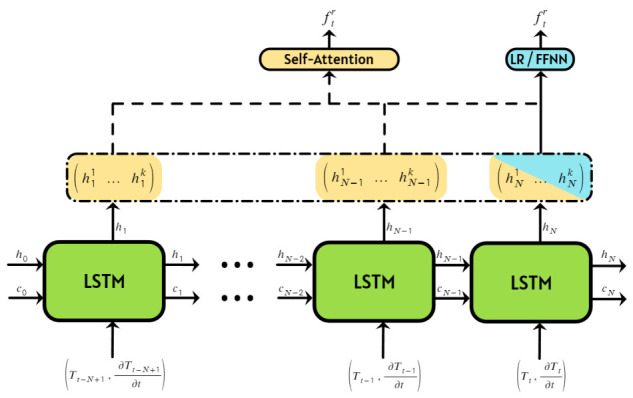
LSTM-based resonance-frequency prediction framework for temperature-dependent PAS sensor stabilization [57]. It is reproduced from ref. [57] with permission from MDPI by CC BY 4.0.

A related but physically distinct strategy is to compensate the temperature-dependent operating state of the sensor rather than correcting the concentration output directly. Borozdin et al. addressed the long-term stabilization of a resonant photoacoustic detector by predicting the gas-cell resonance frequency from temperature-dependent time-series inputs using LSTM-based models [57]. This strategy addresses temperature-induced resonance mismatch, in which changes in gas-cell sound velocity shift the acoustic resonance frequency and thereby weaken the photoacoustic response. As shown in Figure 8, the input sequence consists of the measured temperature and its temporal derivative at successive time steps. In the LSTM module, h_0_ and c_0_ denote the initial hidden state and cell state, respectively, which are recurrently updated to capture the history-dependent thermal evolution of the photoacoustic cell. The self-attention module further assigns different weights to the LSTM hidden states within the temporal window, allowing more informative thermal states to contribute more strongly to the final resonance-frequency prediction. Compared with direct resonance tracking methods that may require additional acoustic excitation or modulation-frequency scanning, this learning-based approach enables continuous resonance-frequency estimation without interrupting laser operation, achieving a mean absolute error below 1 Hz for frequency shifts exceeding 30 Hz over four-hour measurements. This work extends machine learning from concentration-output correction to operating-state stabilization, providing a complementary route for robust photoacoustic sensing under temperature-varying conditions. Overall, different parameter-inversion models should be selected according to the dominant source of uncertainty: feature-enhancement models are suitable for low-SNR signals, optimization-assisted regression models improve prediction robustness under noisy or nonlinear responses, and temporal models are useful when system states vary dynamically during measurement.

### 4.2. Multicomponent Recognition and Interference Decoupling

**Figure 9:**
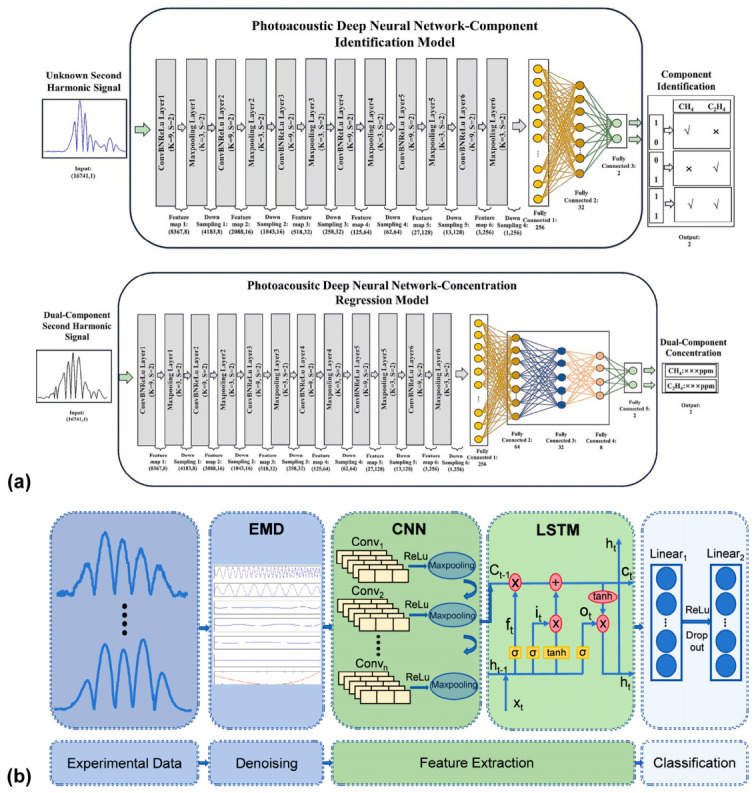
(**a**) Two-stage deep neural network for component identification and concentration regression [62]; (**b**) EMD-CNN-LSTM framework for modal-denoising-assisted multicomponent classification [63]. (**a**) is reproduced from ref. [62] with permission from Elsevier. (**b**) is reproduced with permission from ref. [63]: Copyright American Chemical Society.

Although the above studies mainly improve concentration retrieval and environmental compensation for single or relatively simple sensing targets, practical trace gas detection often involves multicomponent mixtures, where spectral overlap and cross-interference can invalidate conventional peak-amplitude calibration and require more advanced decoupling models. Earlier full-spectrum methods such as PLSR and related linear regression approaches showed that component-specific information is distributed across the whole spectral response rather than being confined to isolated peak amplitudes. These methods provided useful baselines for multigas concentration retrieval and spectral-overlap correction by exploiting the complete spectral or harmonic profile, and they were also used to reduce concentration errors caused by matrix-dependent relaxation effects under calibrated mixture conditions [58,59,60,61]. However, because such linear latent-variable models still rely on predefined statistical projections, recent work has begun to use neural networks to identify gas species and decouple concentrations directly from overlapped harmonic signals. Sun et al. reported a CH_4_/C_2_H_4_ dual-component PAS sensor based on a single 3175 nm mid-infrared ICL and a differential photoacoustic cell. In this system, the absorption profiles and 2*f* signals of CH_4_ and C_2_H_4_ strongly overlap, and the characteristic peaks of the mixed signal shift relative to the single-gas responses, making simple peak-value fitting unreliable [62]. To address this problem, the authors designed two task-specific 1D-CNN models, as shown in Figure 9a. The Photoacoustic Deep Neural Network-Component Identification Model first classifies an unknown 2*f* signal as CH_4_, C_2_H_4_, or a dual-component mixture. For single-gas samples, the concentration is then obtained by polynomial fitting of peak amplitude; for mixed samples, the Photoacoustic Deep Neural Network-Concentration Regression Model directly predicts the CH_4_ and C_2_H_4_ concentrations from the overlapped 2*f* waveform. This strategy combines conventional fitting with neural-network-based waveform decoupling, reducing cross-interference without requiring separate spectral windows for the two gases. The reported MDLs were 0.28 ppm for CH_4_ and 1.56 ppm for C_2_H_4_ in single-component detection, and 8.86 ppm for CH_4_ and 4.55 ppm for C_2_H_4_ in dual-component detection.

However, for overlapped WMS-2*f* signals with strong noise-induced waveform fluctuation, directly learning from the original mixed waveform may make the model sensitive to high-frequency noise and local crosstalk artifacts. In this case, the mixed spectrum needs to be structurally simplified before feature extraction, so that concentration-related peak morphology and sequence-level spectral dependencies can be learned more reliably. Gong et al. addressed this problem with an empirical mode decomposition-convolutional neural network-long short-term memory network (EMD-CNN-LSTM) framework for PAS-based C_2_H_2_/NH_3_ mixture analysis [63]. The method shown in Figure 9b first treats each overlapped WMS-2*f* trace as a one-dimensional sequence and uses empirical mode decomposition to split it into several intrinsic mode functions (IMFs), from high to low frequency. Since the first high-frequency IMFs mainly contain fast fluctuations and noise, different IMF-removal strategies were compared, and removing the first IMF gave the best classification performance. The remaining IMFs were then recombined to form a denoised and structurally simplified 2*f* signal. After this modal reconstruction, CNN layers extracted local harmonic features, such as peak shape, shoulder structures, and local crosstalk-induced variations, while LSTM layers modeled the sequential dependence among different parts of the full 2*f* profile. The final linear layers performed 25-class classification, with each class corresponding to one C_2_H_2_/NH_3_ concentration combination, rather than directly fitting concentration from the raw mixed waveform. This design converts heavily overlapped multicomponent sensing into a modal-denoising-assisted sequence classification problem. After optimization, the model reached a test-set accuracy of 99.89%; in additional measurements under changed system conditions, the concentration errors were 0.092 ppm for C_2_H_2_ and 1.902 ppm for NH_3_.

**Figure 10:**
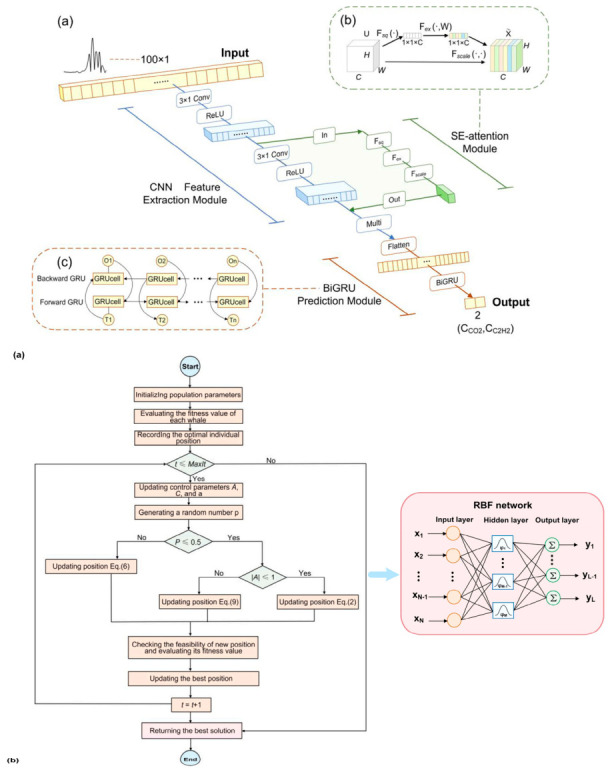
(**a**) Schematic diagram of SSA-CNN-BiGRU-Attention [64]. (**b**) Schematic diagram of WOA-RBF [65]. (**a**) is reproduced with permission from ref. [64]: Copyright American Chemical Society. (**b**) is reproduced from ref. [65] with permission from Elsevier.

More recent LITES studies further extend multicomponent analysis from mixed-signal prediction to selective feature utilization and explicit component-level decoupling. Hou et al. reported a C_2_H_2_/CO_2_ dual-component LITES sensor using an SSA-CNN-BiGRU-Attention model, as shown in Figure 10a [64]. Instead of treating the whole 2*f* waveform uniformly, the model first uses the sparrow search algorithm to automatically optimize training-related parameters, reducing the dependence on manual parameter selection. The CNN module then extracts local morphology from the measured 2*f* signal, including peak, trough, and overlap-induced waveform variations. The BiGRU module further models the bidirectional dependence along the scanned spectral sequence, so that both preceding and subsequent spectral features contribute to concentration prediction. Finally, the attention mechanism assigns larger weights to spectral regions carrying stronger component-specific information, thereby weakening redundant parts of the overlapped 2*f* signal. This framework performs selective nonlinear concentration inversion rather than simple black-box regression. The test-set R^2^ values were all higher than 0.99, and the MRE remained below 1.2% under different spectral-overlap conditions. In a subsequent study, Hou et al. moved one step further from attention-assisted concentration inversion to explicit spectral-line separation for C_2_H_2_/NH_3_ LITES [65]. The problem here is that the mixed 2*f* signal contains more information than concentration values alone, but direct inversion cannot reveal how each gas contributes to the overlapped spectrum. To address this, the authors used the overlapped C_2_H_2_/NH_3_ 2*f* signals as inputs and the experimentally measured unmixed C_2_H_2_ and NH_3_ 2*f* profiles as target outputs, so that the model learned to reconstruct the complete single-gas spectral profiles from the mixed waveform. In Figure 10b, the radial basis function neural network provides nonlinear local approximation through Gaussian basis functions, which is suitable for mapping distorted overlapped spectra to component-specific 2*f* signals. The whale optimization algorithm is used to search for the optimal spread parameter of the RBF network, improving the balance between local fitting accuracy and generalization. After separation, the recovered C_2_H_2_ and NH_3_ 2*f* profiles can be directly compared with true unmixed spectra and then used for concentration fitting. The separated signals showed an MAE below 9.48 × 10^−5^ mV and MRE below 0.2%, with R^2^ > 0.99 for the separated 2*f* amplitudes and an MDL of 0.541 ppm for C_2_H_2_ and 1.350 ppm for NH_3_.

**Figure 11:**
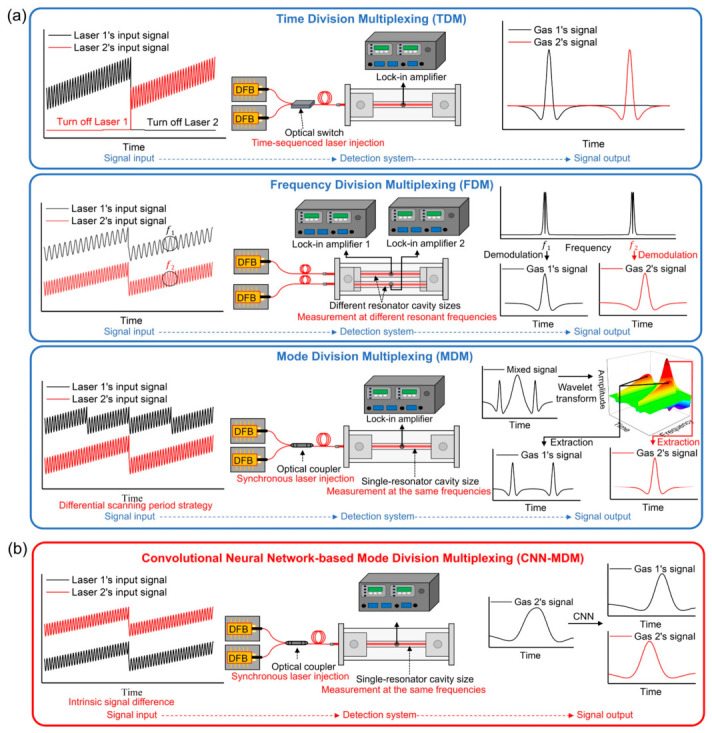
Schematic diagram of CNN-based mode division multiplexing [66]. It is reproduced with permission from ref. [66]: Copyright American Chemical Society.

A further development is the convolutional neural network-based mode division multiplexing (CNN-MDM) strategy for photoacoustic multigas sensing proposed by Liang et al., which further moves multicomponent analysis from post-acquisition spectral decoupling toward modulation-level waveform coding and learning-based demultiplexing [66]. In Figure 11, unlike time-division multiplexing, which sacrifices simultaneity, or frequency-division multiplexing, which requires different detection frequencies and increases system complexity, CNN-MDM enables different gases to be measured at the same acoustic resonant frequency. In the CO/H_2_S demonstration, two lasers were used for the two gases, while both channels were modulated at 349 Hz so that their 2*f* photoacoustic signals were detected at the same 698 Hz resonance of the photoacoustic cell. Different low-frequency scanning periods were then assigned to CO and H_2_S to generate gas-specific waveform modes. For training, pure CO and H_2_S signals were amplitude-scaled, noise-augmented, and superimposed to synthesize mixed inputs, while the corresponding pure component signals served as target outputs. A one-dimensional CNN learned local waveform features from the mixed signal and separated it into CO- and H_2_S-related signal components, which were then used for concentration quantification. The separated signals showed strong linearity with concentration, with R^2^ values of 0.996 and 0.995 for CO and H_2_S, and MDLs of 50 ppb and 426 ppb, respectively. Overall, these methods differ in the level at which multicomponent interference is addressed: classification-based models mainly determine gas identity or concentration categories from distorted spectra, sequence- and attention-based models learn concentration-related features distributed across overlapped harmonic profiles, whereas signal-separation and demultiplexing models aim to reconstruct component-specific responses from mixed measurements before quantitative analysis.

In parameter inversion, wavelet-assisted feature extraction, CNN-type regression, and optimization-enhanced ensemble models improve concentration retrieval under a low SNR, source fluctuation, and nonlinear response. For environment-affected sensing, intelligent compensation models correct response drift induced by temperature or operating-condition changes. For multicomponent sensing, deep-learning models further enable gas recognition, cross-interference suppression, overlapped waveform separation, and modulation-level waveform demultiplexing. These studies indicate that intelligent inversion is evolving from simple concentration fitting toward integrated quantitative analysis, interference correction, and signal decoupling.

## 5. Intelligent System Design and Optimization

### 5.1. Intelligent Optimization of PACs

**Figure 12:**
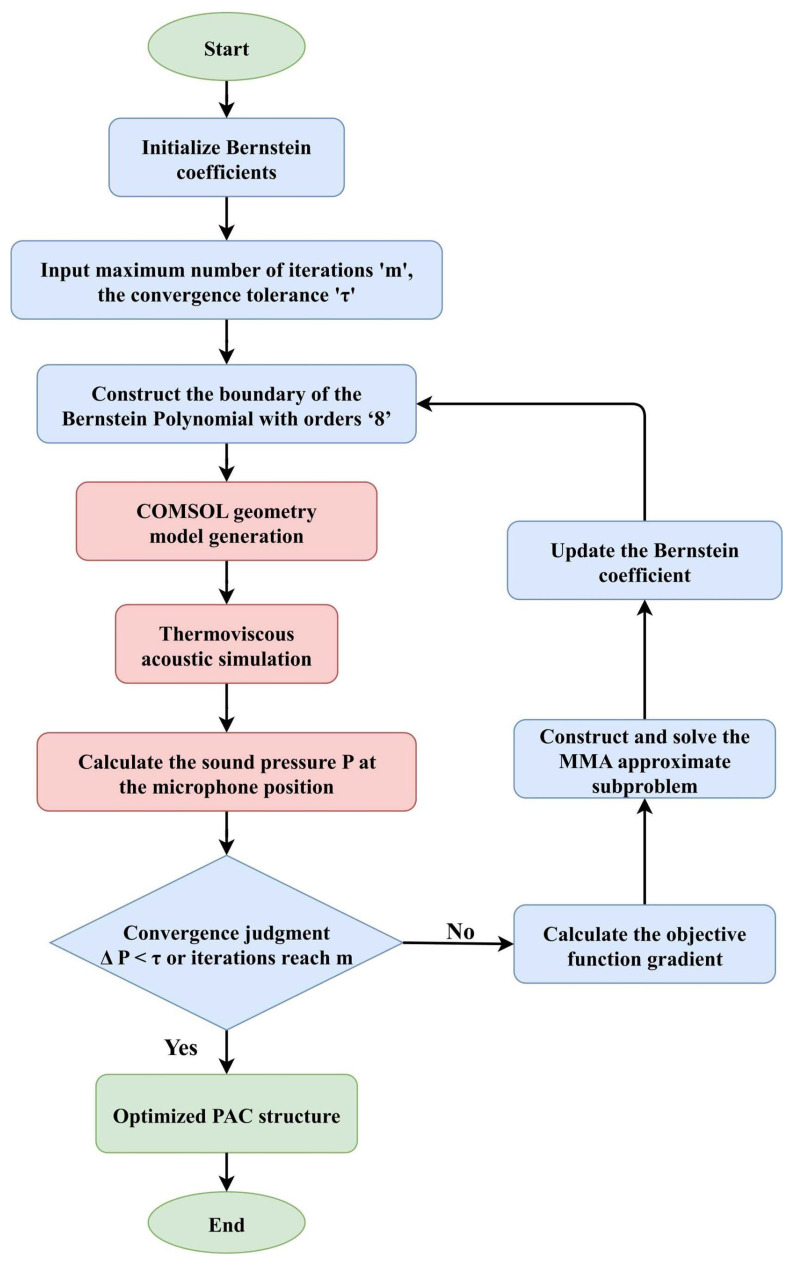
Topology optimization of PAC based on MMA-BP [67]. It is reproduced from ref. [67] with permission from Elsevier.

Beyond recovering measured signals and extracting information, intelligent algorithms are also being extended to the front-end design stage, where the physical structures of sensing systems are optimized to improve the original signal strength, stability, and information quality. PAC optimization is constrained by the nonlinear coupling among resonator geometry, acoustic-mode distribution, Q factor, and cell volume. In miniaturized cells, empirical dimension tuning or one-factor parameter sweeping can hardly explore the full design space, because small geometric variations may reshape the acoustic mode and shift the pressure maximum away from the microphone. To address this shape-search problem, Wang et al. proposed an MMA-BP topology optimization method for photoacoustic resonators shown in Figure 12, in which Bernstein polynomials were used to parameterize the resonator boundary and the method of moving asymptotes iteratively updated the shape variables to maximize the acoustic pressure response. During the optimization process, several representative PAC geometries were compared, including the initial T-PAC, trapezoidal resonator PAC, microcone-curved resonator PAC, and finally, the optimized vase-type PAC [67]. The final V-PAC had a compact volume of 5 mL and achieved a C_2_H_2_ MDL of 281 ppt at 768 s, corresponding to an NNEA of 4.46 × 10^−9^ cm^−1^ Hz^−1/2^ with a single optical path; compared with a conventional T-PAC, the overall performance was improved by approximately 14 times.

**Figure 13:**
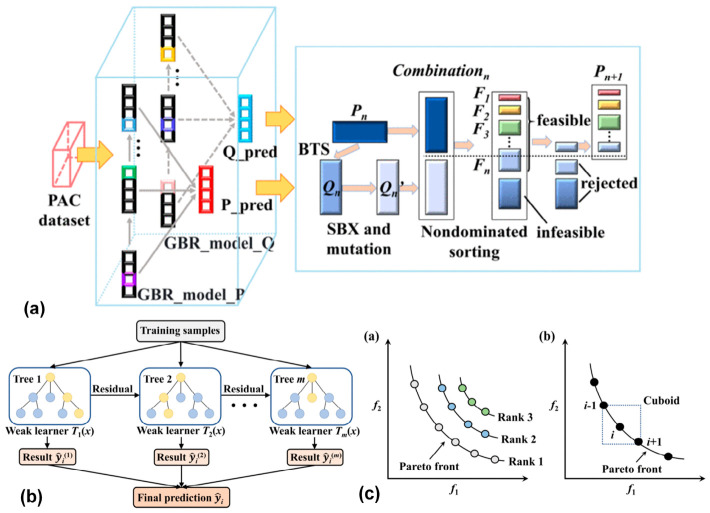
Surrogate-model-assisted multi-objective optimization of a T-type PAC: (**a**) overall GBDT–NSGA-II framework [68]; (**b**) principle of GBDT-based surrogate model [68]; and (**c**) NSGA-II selection mechanism based on nondominated sorting and crowding-distance preservation [68]. (**a**–**c**) are reproduced from ref. [68] with permission from the IEEE.

When PAC geometry involves multiple design variables and competing objectives, exhaustive finite-element scanning becomes inefficient. Zhu et al. proposed a GBDT–NSGA-II framework for optimizing a truncated cone–double petal T-type photoacoustic cell, as shown in Figure 13a [68]. Finite-element simulations first generated a dataset linking seven structural parameters to acoustic pressure and Q-factor, and two GBDT surrogate models were then trained separately to predict these two responses, replacing repeated FEA evaluations during optimization. In the GBDT model shown in Figure 13b, the geometry–response relationship is learned as an additive ensemble of regression trees: the first tree gives an initial prediction, and each subsequent tree fits the residual error, or negative gradient, left by the previous ensemble, so that nonlinear mapping accuracy is progressively improved under limited simulation samples. NSGA-II then used the surrogate-predicted pressure and Q-factor as dual objectives. Candidate geometries were generated through selection, simulated binary crossover, and mutation; the parent and offspring populations were merged and ranked by nondominated sorting and crowding-distance calculation, as shown in Figure 13c. This elitist preservation strategy retained high-ranking Pareto solutions while maintaining diversity along the trade-off front, and infeasible or crowded inferior solutions were rejected when forming the next generation. As a result, the optimization balanced pressure enhancement and resonance quality, with prediction errors of only 0.27% for acoustic pressure and 0.70% for Q-factor. The optimized TCDPT-PAC achieved a C_2_H_2_ sensitivity of 20.48 pm/ppm, 2.95 times that of a conventional H-type cell, and an MDL of 2.93 ppb at 100 s.

### 5.2. Intelligent Optimization of MPCs

**Figure 14:**
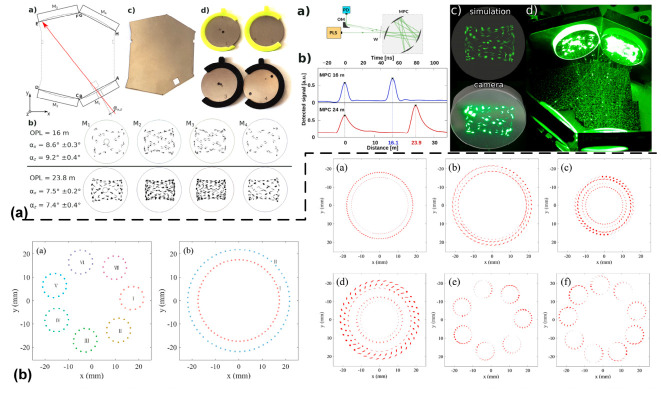
(**a**) GA-based dense astigmatic spot patterns using standard spherical mirrors [69]. (**b**) PSO–K-means-based concentric-circle spot-pattern optimization [70]. (**a**,**b**) are reprinted from ref. [69] and [70], respectively, with permission from Optica Publishing Group.

MPC optimization is not merely a search for the longest optical path; it must also balance compact volume, mirror utilization, spot regularity, non-overlap, and alignment feasibility. Early algorithm-assisted studies addressed this coupled optical-design problem by replacing empirical mirror adjustment with global search. Hudzikowski et al. used a genetic algorithm to design a compact spherical-mirror MPC with dense astigmatic-like spot patterns, experimentally realizing 16 m and 23.8 m optical paths and achieving a 0.4 ppmv CO_2_ detection limit with the 23.8 m MPC, as shown in Figure 14a [69]. Kong et al. integrated K-means clustering into PSO for the intelligent design of two-spherical-mirror MPCs [70]. In this framework, PSO searches the geometrical and injection parameters of the cell, whereas K-means is used to evaluate the regularity of simulated circular spot patterns, enabling simultaneous optimization of long optical path length and well-organized mirror spot distributions. In Figure 14b, the algorithm generated six optimized circular-spot candidate configurations with optical path lengths exceeding 50 m, from which a representative four-concentric-circle MPC was experimentally constructed, achieving a 54.1 m optical path length within a 273.1 cm^3^ volume and enabling CH_4_ detection with an 8 ppb precision at 13 s averaging time.

**Figure 15:**
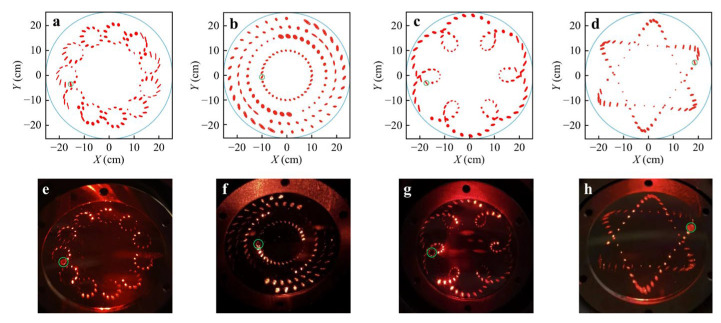
Simulated and measured dense spot distributions on the mirrors [71]. It is reproduced from ref. [71] with permission from Light Publishing Group of CIOMP by CC BY 4.0.

In this way, MPC optimization began to move from simply extending optical path length toward simultaneously screening ordered, non-overlapped, and experimentally alignable spot distributions. A reliable ray-tracing model is the prerequisite for such algorithmic optimization, especially for dense-spot MPCs operating beyond the paraxial approximation of conventional Herriott-cell theory. Ma et al. therefore developed a vector-reflection-based ray-tracing model for dense spot-pattern MPC design [71]. In this model, each reflected beam direction and spot coordinate were iteratively calculated using the vector form of the reflection law, while the incident position, incident angles, mirror spacing, and curvature radius were tuned under constraints on spot overlap and optical-path-to-volume ratio. Four dense spot-pattern MPCs were obtained as shown in Figure 15 and experimentally verified, including independent-ring, four-concentric-circle, flower, and six-pointed-star patterns. Among them, the four-concentric-circle MPC achieved an actual optical path length (OPL) of 38.1 m and a ratio of optical path length to volume (RLV) of 13.8 cm^−2^, and was applied to CH_4_-LITES detection. This work provides the computational basis on which later swarm-intelligence and multi-objective MPC optimization can be built.

**Figure 16:**
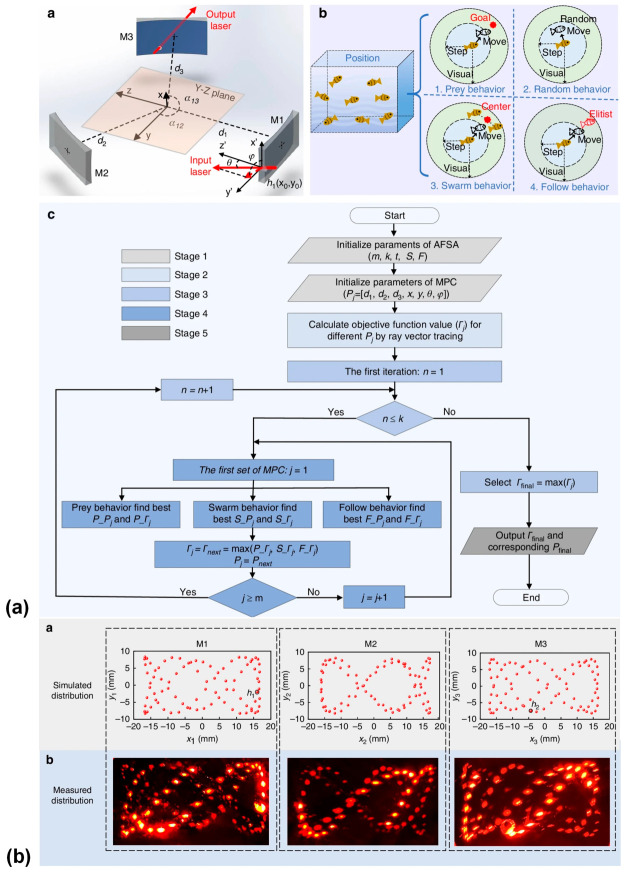
(**a**) The framework of artificial-fish-swarm-algorithm-assisted design of a three-mirror MPC [72]; (**b**) simulated and measured dense spot distributions on the mirrors [72]. (**a**,**b**) are reprinted from ref. [72] with permission from Springer Nature by CC BY 4.0.

Building on this modeling basis, Sun et al. introduced an artificial fish swarm algorithm (AFSA) to automate the design of a three-mirror MPC with a double-helix spot pattern for CO-LITES [72]. The motivation was that adding a third mirror increases the dimensionality of the optical system, making manual tuning of mirror distances, incident position, and incident angles inefficient. As shown in Figure 16a, each artificial fish represented a candidate optical geometry, encoded as *P_j_*. For each candidate, vector ray tracing calculated the reflection trajectory, reflection number, OPL, and cell volume. The objective function Γ = *ω*1OPL + *ω*2OPL/V balanced absorption length and compactness, while a reflection-number constraint limited transmission loss from the 98% reflective mirrors. During iteration, prey, swarm, follow, and random behaviors updated candidate geometries, and the solution with the highest objective value was retained. In Figure 16b, the optimized MPC produced a double-helix spot pattern with 259 reflections, an OPL of 25.8 m, a volume of 165.8 mL, and an OPL/V of 15.6 cm^−2^. Combined with a PDMS-modified low-frequency round-head QTF, the CO-LITES sensor achieved an MDL of 23 ppt, which further improved to 920.7 ppq at 500 s averaging time.

**Figure 17:**
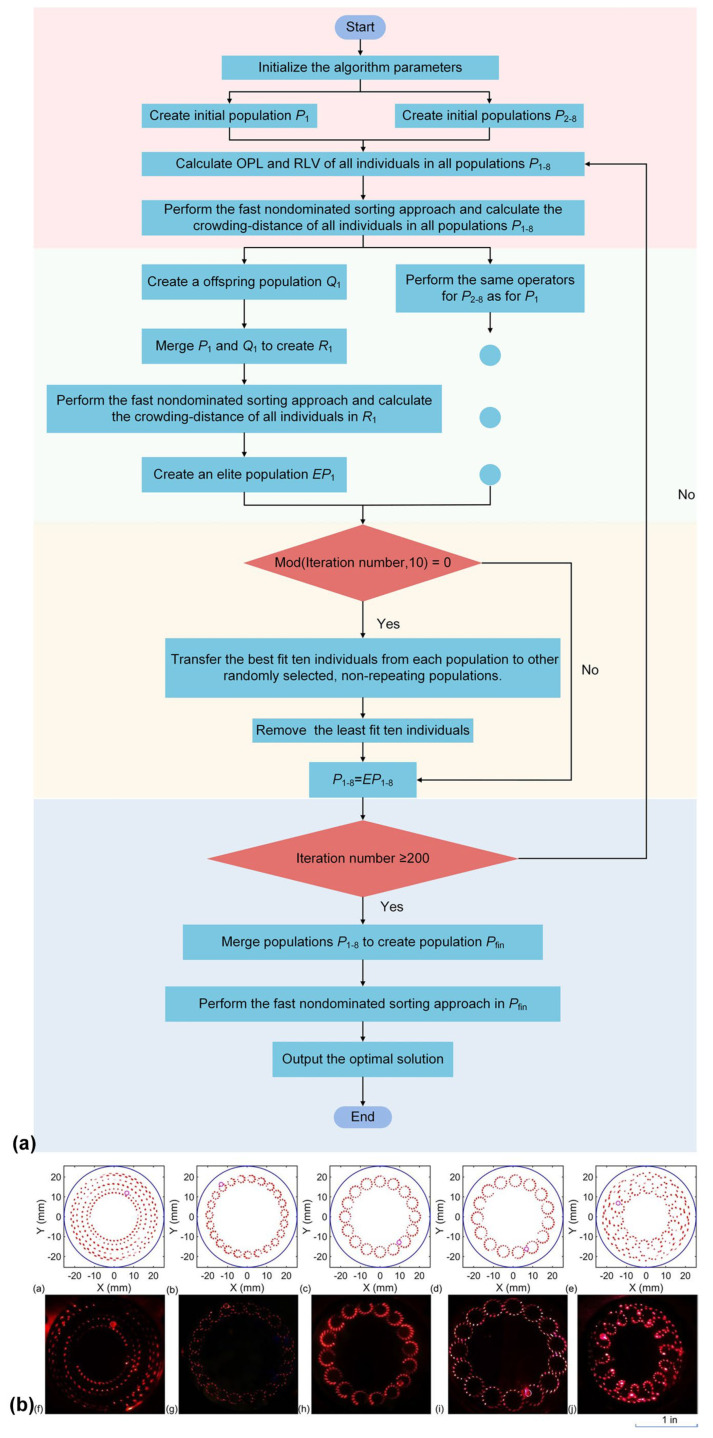
(**a**) Parallel nondominated sorting genetic algorithm II for multi-objective MPC optimization [73]; (**b**) simulated and measured dense spot distributions on the mirrors [73]. It is reprinted from ref. [73] with permission from Springer Nature by CC BY 4.0.

More recently, MPC design has moved from weighted-objective single-configuration search toward Pareto-based multi-objective optimization. Ma et al. employed a parallel nondominated sorting genetic algorithm II, PNSGA-II, to design ultra-dense spot-pattern MPCs for LITES [73]. In Figure 17a, each individual represents a candidate MPC geometry encoded by mirror distance, incident point, and incident angles (*d*, *x*_0_, *y*_0_, *θ*, *φ*), while vector ray tracing calculates the corresponding spot distribution, OPL, and RLV. Unlike weighted-objective strategies that merge OPL and compactness into a single fitness function, PNSGA-II treats OPL and RLV as independent objectives to obtain Pareto-optimal MPC candidates. Multiple populations are evolved in parallel; within each population, selection, simulated binary crossover, and mutation generate offspring, while fast nondominated sorting and crowding-distance calculation preserve Pareto-optimal candidates with both a high absorption length and compact volume. A migration operator periodically transfers the best individuals among populations, improving global exploration and reducing runtime. As shown in Figure 17b, the algorithm generated five ultra-dense spot-pattern MPCs, from which the fifteen-ring-cluster design was selected for sensing. This MPC achieved an actual OPL of 80.14 m and RLV = 22.17 cm^−2^, and the corresponding C_2_H_2_-LITES sensor reached an MDL of 4.78 ppb, further improved to 891 ppt at 200 s averaging time.

Collectively, intelligent system design and optimization in PAS-, QEPAS-, and LITES-based sensing have mainly advanced along two routes: acoustic-structure optimization and optical-path design. For PACs, topology optimization, surrogate modeling, and multi-objective search have enabled more systematic exploration of resonator geometry, acoustic-pressure enhancement, resonance quality, and miniaturization. For MPCs, the design strategy has evolved from empirical optical alignment to ray-tracing-based modeling, intelligent search, spot-pattern evaluation, and multi-objective optimization, thereby improving the balance among optical path length, compactness, spot distribution, and experimental feasibility. In general, topology-optimization methods are suitable for exploring non-intuitive acoustic-cell geometries under physical constraints, surrogate-model-assisted optimization is effective for reducing repeated finite-element simulation cost, and swarm-intelligence or multi-objective algorithms are more appropriate when several coupled design targets need to be balanced simultaneously. These studies show that sensor-structure design is gradually moving from experience-driven parameter adjustment and repeated simulation trials toward model-guided design, algorithm-assisted optimization, and closed-loop experimental validation.

## 6. Challenges and Perspectives

Future intelligent PAS-, QEPAS-, and LITES-based sensing should move from isolated offline algorithms toward physically constrained, closed-loop, and field-deployable sensing systems. For spectral reconstruction, reliable clean references remain difficult to obtain, because long-term averaging, repeated measurements, and paired acquisition can be distorted by laser drift, concentration fluctuation, temperature variation, and system instability, causing supervised models trained on averaged processed “clean” spectra to inherit reference bias. As such, unsupervised or self-supervised reconstruction should be further explored to reduce dependence on ideal labels [74,75,76]. Signal processing should also extend beyond post-acquisition denoising by jointly considering modulation strategy, lock-in demodulation, averaging strategy, waveform coding, and learning-based reconstruction, thereby linking front-end signal design with back-end intelligent inference. For concentration inversion and multicomponent sensing, models trained under fixed devices, gas matrices, or operating conditions may degrade under temperature and pressure variations, optical fringes, QTF resonance drift, electronic noise, and long-term drift, particularly in the presence of spectral overlap. Future models should therefore integrate data-driven learning with physical priors, environmental compensation, and transfer learning. Physical consistency and interpretability should be strengthened by embedding optical, acoustic, electromechanical, and concentration-response constraints into model architectures, loss functions, or output regularization, promoting physics-informed learning for sensing [77,78]. Residual statistics, uncertainty estimation, and ablation studies should be used to assess whether learned features are physically meaningful and prediction reliability is quantifiable [79,80]. At the system-design level, intelligent optimization should evolve from component-level tuning to closed-loop co-design of optical, acoustic, and electromechanical modules. AI-assisted inverse design is a particularly promising direction, where target sensing responses guide the generation of feasible PACs, resonators, QTFs, MPCs, and coupling structures under constraints of manufacturability, numerical validity, and experimental performance [81,82]. Finally, model deployment remains insufficiently explored. Future studies should report computational cost, inference latency, model size, embedded compatibility, online drift correction, and adaptive calibration, enabling intelligent methods to progress toward real-time and field-deployable gas sensing [83,84].

## 7. Conclusions

This review summarizes recent progress in intelligent PAS-, QEPAS-, and LITES-based systems for trace gas sensing. Recent studies show that algorithm-assisted methods are being incorporated into three key layers of these sensing systems: signal processing and spectral reconstruction, parameter inversion and complex spectral analysis, and intelligent system design and optimization. These layers address weak-signal recovery, reliable quantitative analysis, and efficient sensor-structure design. In signal processing and spectral reconstruction, recent studies have moved from traditional signal-processing algorithms toward more efficient and higher-performance AI-based adaptive signal recovery, further enabling nonlinear noise suppression, structured-background removal, and harmonic-profile reconstruction. In parameter inversion and complex spectral analysis, AI models and intelligent algorithms have improved the capability for concentration retrieval, environmental compensation, multicomponent recognition, overlapped-signal decoupling, and front–back-end collaborative waveform coding and demultiplexing. In system design, surrogate modeling, swarm-intelligence search, physics-model-guided topology optimization and multi-objective optimization have begun to enhance the design efficiency of PACs and MPCs in an intelligent manner. Taken together, intelligent algorithms are shifting from auxiliary post-processing tools toward broader participation in the sensing chain. With the deepening integration of artificial intelligence, intelligent optimization, and laser spectroscopic sensing, gas sensing is expected to evolve toward higher reliability, stronger adaptability, higher quantitative accuracy, and more practical deployment in trace gas detection.

## Data Availability

The data presented in this study are available on request from the corresponding authors.
